# Technology-enabled virtual ward for COVID management of the elderly and immunocompromised in Singapore: a descriptive cohort

**DOI:** 10.1186/s12879-023-08040-2

**Published:** 2023-02-21

**Authors:** Stephanie Q. Ko, Shoban Krishna Kumar, Jonathan Jacob, Benjamin M. Y. Hooi, Michelle Soo, Norshima Nashi, Maria Teresa D. Cruz, Yeo Ai Wah, Wong Zhi Xin, Nares Smitasin, Lionel Lum, Sophia Archuleta

**Affiliations:** 1 Division of Advanced Internal Medicine, Department of Medicine, National University Hospital, 1E Kent Ridge Road, Singapore, 119228 Singapore; 2 Department of Medicine, Alexandra Hospital, 378 Alexandra Road, Singapore, 159964 Singapore; 3 Regional Health Service, National University Health System, 1E Kent Ridge Road, Singapore, 119228 Singapore; 4 Division of Infectious Diseases, Department of Medicine, National University Hospital, 1E Kent Ridge Road, Singapore, 119228 Singapore

**Keywords:** COVID-19, Hospital-at-home, Virtual Ward

## Abstract

**Background:**

To address the hospital bed demand for Delta and Omicron surges in Singapore, the National University Health System (NUHS) developed a COVID Virtual Ward to relieve bed pressures on its three acute hospitals—National University Hospital, Ng Teng Fong General Hospital, Alexandra Hospital. To serve a multilingual population, the COVID Virtual Ward featuring protocolized teleconsultation of high-risk patients, use of a vital signs chatbot, supplemented by home visits where necessary. This study aims to evaluate the safety, outcomes and utilisation of the Virtual Ward as a scalable response to COVID-19 surges.

**Methods:**

This is a retrospective cohort study of all patients admitted to the COVID Virtual Ward between 23 September to 9 November 2021. Patients were defined as “early discharge” if they were referred from inpatient COVID-19 wards and “admission avoidance” if they were referred directly from primary care or emergency services. Patient demographics, utilisation measures and clinical outcomes were extracted from the electronic health record system. The primary outcomes were escalation to hospital and mortality. Use of the vital signs chatbot was evaluated by examining compliance levels, need for automated reminders and alerts triggered. Patient experience was evaluated using data extracted from a quality improvement feedback form.

**Results:**

238 patients were admitted to the COVID Virtual Ward from 23 September to 9 November, of whom 42% were male, 67.6% of Chinese ethnicity. 43.7% were over the age of 70, 20.5% were immunocompromised, and 36.6% were not fully vaccinated. 17.2% of patients were escalated to hospital and 2.1% of patients died. Patients who were escalated to hospital were more likely to be immunocompromised or to have a higher ISARIC 4C-Mortality Score. There were no missed deteriorations. All patients received teleconsults (median of 5 teleconsults per patient, IQR 3–7). 21.4% of patients received home visits. 77.7% of patients engaged with the vital signs chatbot, with a compliance rate of 84%. All patients would recommend the programme to others in their situation.

**Conclusions:**

Virtual Wards are a scalable, safe and patient-centered strategy to care for high risk COVID-19 patients at home.

***Trial Registration*:**

NA.

**Supplementary Information:**

The online version contains supplementary material available at 10.1186/s12879-023-08040-2.

## Background

Singapore’s COVID-19 pandemic began with the identification of its 1st imported case on 23rd January 2020 [1] and 1st local transmission on 4th February 2020 [2]. Since then, Singapore has gone through several waves of COVID-19 infection with a total of 274, 972 cases as of 17th December 2021 [3]. The healthcare system’s response and corresponding public health measures required rapid adaptation to each surge—the most significant occurring during the peak of the Delta wave in late 2021.

The initial containment approach to COVID-19 required all patients to be admitted and isolated in a healthcare facility, regardless of symptoms or severity of illness, until they were deemed no longer infectious. Community facilities were set up to house COVID-19 patients with mild symptoms to ease hospital occupancy rates [4]. However, with the emergence of the Delta variant and a peak of 3000–5000 daily infections [3], institutionalizing all COVID-19 patients presented an unprecedented strain on healthcare resources [3, 5].

In alignment with the nation’s transition toward a COVID-19 endemic state, the Home Recovery Programme (HRP) was implemented by the Ministry of Health in September 2021 for fully vaccinated individuals aged 12–50 with no severe comorbidities [6]. Later, this programme expanded to include fully vaccinated individual up to 80 years old and unvaccinated individuals up to 50 years old. Patients who did not meet these criteria, predominantly older adults and patients with comorbidities, were deemed higher risk and continued to be admitted to a healthcare facility.

Remote home monitoring models have been reported to be a safe and effective strategy to manage patients with COVID-19 [7] in the USA [8–13], UK [14, 15] and China [16] whilst utilizing oximeters to detect at risk patients. Most models have focused on low to moderate risk patient groups which in Singapore can be cared for under the HRP model. However, hospitalisation of older adults are often associated with high rates of functional decline and hospital acquired infections [17].

Immunocompromised patients especially are at risk and also subject to longer hospital stays due to poor vaccine immunogenicity and also subject to a longer hospital stays due to a longer infectivity period. In response to the urgent need for home-based care of these higher-risk patients, our hospital’s existing hospital-at-home service opened a COVID virtual ward in September 2021, after a period of 2 weeks of preparation.

The aim of the virtual ward is to provide comprehensive care at home to substitute the hospital stay thereby avoiding hospital admissions (“admission avoidance”) [18, 19] or allowing earlier discharge from healthcare facilities (“early discharge”) [15, 20]. The use of remote monitoring and teleconsultation enables prompt escalation of at risk cases for early treatment (e.g. monoclonal antibodies), early identification of deterioration to avoid intubation [21] and management of chronic diseases during the isolation period.

To understand whether the virtual ward was effective and should be scaled for future responses to COVID-19 surges, we conducted a retrospective cohort study to evaluate the safety and outcomes of patients under the Virtual Ward care, and describe the utilisation of teleconsultation, home visits and vital signs monitoring.

## Methods

### Program description

#### Setting

The National University Health System (NUHS) is an integrated healthcare system in the Western region of Singapore, comprising three acute hospitals—National University Hospital, Ng Teng Fong General Hospital, Alexandra Hospital and several primary care clinics (polyclinics). The COVID Virtual Ward is run by NUHS’ existing Hospital-at-Home Programme, NUHS@Home.

Patients were referred to the COVID Virtual Ward from two sources. First, COVID-19 inpatient wards at all three hospitals may refer hospitalized patients once stable. Such patients would have received initial treatment in a hospital ward, then transferred to the Virtual Ward for the rest of their isolation period, thereby reducing their hospital length-of-stay. We refer to this cohort as the “early discharge” cohort. Second, primary care practices, outpatient clinics and emergency departments may refer patients identified to have COVID-19 but are not yet admitted to the hospital. These patients would be admitted directly to the Virtual Ward rather than being admitted to a hospital ward at all. We refer to this cohort as the “admission avoidance” cohort.

Patients who were eligible for HRP were excluded from the COVID Virtual Ward. Differences between HRP and COVID Virtual Ward are summarized in Table 1.

**Table 1:** Differences between home recovery programme and COVID virtual ward

	Home Recovery Programme	COVID Virtual Ward
Governing body	Ministry of Health operational arm	Hospital clinical team and clinical governance
Target population	Unvaccinated < 50 years old	Unvaccinated ≥ 50 years old
	Vaccinated < 80 years old	Vaccinated ≥ 80 years old
	No comorbidities	Any comorbidities (except haemodialysis)
Care model	Patient responsibility to self-monitor and escalate if unwell (recommended once daily vitals)	Clinician-supervised service Enforced vitals up to three times daily during critical period
Aims	Safe admission avoidance for low risk patients	Reliable deterioration recognition for high risk adults
		Management of chronic disease during unwell and isolation period
Clinical contact	Teleconsult once after enrolment and as requested by patient	Teleconsult daily or every other day
		Home visits as required (e.g. blood tests, PICC flushes)

#### Patient selection

Referral criteria were clinical stability (did not require oxygen, no symptoms of breathlessness); not on haemodialysis; wished to be cared for at home. Patients who met prevailing criteria for HRP [6] were excluded. There were no limitations on geographical boundaries of patients’ place of residence.

All referrals were screened by COVID Virtual Ward doctors over teleconsultation for clinical stability, home suitability (satisfactory ability to self-isolate from vulnerable household members), and ability to engage with teleconsultations and self-monitor vital signs. Eligible patients were then admitted to the COVID Virtual Ward.

#### Treatment protocol

The COVID Virtual Ward treatment protocol was developed taking into account patients’ day of illness, clinical risk, vaccination status and presence of immunocompromised state. Management includes regular teleconsultations by nurses, scheduled teleconsultations by doctors, teleconsultations by pharmacist where required, and regular vital signs monitoring. During the critical phase, defined as day 1 to 5 from symptom onset for fully vaccinated individuals and day 1 to 8 from symptom onset for not fully vaccinated or immunocompromised individuals, patients received teleconsultation daily combined with a modified clinical examination, the 1 min Sit-To-Stand test [22–24] to determine exertional hypoxia, with vital signs monitored three times daily. In the recovery phase, the teleconsultation frequency was reduced to every other day and vital signs to twice daily. For immunocompromised individuals in the last week of isolation, teleconsultation frequency was as needed only, with vital signs once daily (Additional file 1: Fig. S1).

During the treatment period, a private medical house call agency assisted with nursing visits for blood tests, patient review or nursing procedures (e.g. wound dressing, PICC flushing). If further medication top up or adjustment was required, the medication delivery and counselling via teleconsultation were arranged by the Virtual Ward pharmacist. Treatment protocols were individualized according to other patient needs, for example laboratory tests were repeated weekly for transplant patients, and patients on warfarin had their INR monitored as required.

#### COVID-19 therapeutics

Prevailing national guidance for administration of the recommended monoclonal antibody sotrovimab [26] were: (1) within day 1–5 of illness; (2) had an insufficient spike antibody response; and (3) had an elevated ISARIC 4-C mortality score [25]. For patients in the early discharge cohort, patients would have been risk stratified and monoclonal antibodies administered prior to transfer to the Virtual Ward. For patients in the admission avoidance cohort, we routinely performed blood tests upon enrolment including COVID serology, urea and C-reactive protein to calculate the ISARIC 4-C mortality score, and escalate patients back to the hospital for early treatment if they met criteria (Additional file 1: Fig. S2). Such patients were transferred back to the Virtual Ward after antibody administration if well and were included as being escalation to hospital for monoclonal antibodies.

#### Vital signs chatbot

Patients were provided with monitoring equipment as required (e.g. thermometer, blood pressure machine or oximeter). The care team educated patients on how to monitor their own vital signs at the start (blood pressure, oxygen levels and temperature). The preferred method of vital signs monitoring was a chatbot that sent an online reporting form to patient’s (or caregiver’s) mobile in their preferred language (English, Mandarin or Malay). Patients (or caregivers) entered the vital signs into the form, which are uploaded into an online clinician dashboard. If patients did not submit their vital signs by the designated timing (e.g. 8am, 2 pm, 8 pm for three times daily parameter reporting), they would receive an automated push notification to remind them to do so. If they did not report within 2 h, a clinician on the team would reach out to them to ensure that they are well. If the readings exceeded pre-set thresholds (temperature ≥ 38 °C, SpO_2_ < 95%, heart rate > 100 beats per minute or < 50 beats per minute, systolic blood pressure > 180 mmHg or < 100 mmHg, diastolic blood pressure > 100 mmHg or < 60 mmHg, the clinicians would receive a SMS-based alert for follow up. If patients or caregivers were not able to engage with this automated system, the team would reach out to them via text messaging platforms or phone calls at the designated timings to receive their vital signs readings.

#### Escalation and discharge

During the treatment period, if patients developed low saturations, concerning new symptoms, or required treatment with monoclonal antibodies, they were transferred back to the hospital for further treatment and assessment.

Patients were discharged from COVID Virtual Ward care when clinically well, and were no longer required to be isolated by prevailing government guidelines (14 days for not fully vaccinated, 10 days for fully vaccinated, and 21 days for immunocompromised patients, from date of symptom onset). The cost of the COVID Virtual Ward admission were borne by the Ministry of Health in accordance to prevailing policies [4].

#### Staffing

The COVID Virtual Ward was staffed according to the following ratios: 1 consultant:100 patients, 1 junior doctor:15 patients and 1 nurse:25 patients during office hours, and 1 junior doctor:100 patients after office hours.

### Study design and data collection

A retrospective audit of all patients admitted to the COVID Virtual Ward from 23 September to 9 November 2021 was conducted.

Patient demographics, utilisation measures and clinical outcomes were extracted from the electronic health record system. Demographic measures included day of illness, immunocompromised status and reason for immunocompromised status, vaccination status, and ISARIC-4C Mortality Score [25]. The ISARIC-4C Mortality Score is an in-hospital mortality score that incorporates age, sex, number of comorbidities (chronic cardiac disease, chronic respiratory disease excluding asthma, chronic renal disease as defined by estimated glomerular filtration rate ≤ 30, mild to severe liver disease, dementia, chronic neurological conditions, connective tissue disease, diabetes mellitus, HIV or AIDS, and malignancy), respiratory rate, oxygen saturation on room air, Glasgow Coma Scale, Urea and C-reactive protein (CRP). If patients did not have laboratory investigations available, they were assumed to have Urea and CRP within normal limits. Patients were considered immunocompromised if they had: an organ transplant, cancer on chemotherapy, a haematological malignancy, or non-cancer conditions who requiring immunosuppressive treatment.

The primary outcomes included escalation to hospital, escalation to hospital for COVID-related reasons, and death. Escalation to hospital for COVID-related reasons was defined as returns to hospital for hypoxia or for non-hypoxic COVID-related reasons (e.g. prolonged fever, functional decline, diarrhoea). Death included patients who passed on during the virtual ward stay and those who were escalated to hospital and subsequently passed on. Utilisation outcomes included length of stay in the COVID Virtual Ward, home visits conducted, swabs performed, blood tests done, number of teleconsultations conducted and whether they were scheduled or unscheduled, method of vital signs monitoring and number of courier trips required.

Utilisation of the vital signs chatbot was measured by extracting data from the vital signs dashboard. Compliance was determined by both the proportion of readings for each patient that required reminders, and the proportion of overall reminders that triggered a patient submission. Clinical alerts were described as proportion of patients who triggered an alert, and those for elevated temperature or low oxygen saturations.

Anonymous data was extracted from an online quality improvement survey sent to all patients post-discharge.

### Sample size

All patients admitted during the study period were included. The outcomes of this study would be used to determine whether the COVID Virtual Ward programme should be maintained and scaled.

### Statistical analysis

Descriptive statistics were used to analyse patient characteristics and health outcomes. Categorical variables are presented as frequencies and percentages. Continuous variables are presented as means with standard deviations for parametric variables and medians with interquartile ranges for non-parametric variables. Differences in demographics between early discharge and admission avoidance cohorts were compared using chi-square tests for categorical variables, two-sided t-tests for continuous parametric variables and the Wilcoxon-rank test for continuous non-parametric variables. Difference in demographics between patients who were escalated to hospital and those who were not were also compared using similar techniques.

### Ethics

This study was approved by the National Healthcare Group Domain Specific Review Board (Ref 2021/00896). As this was a retrospective study of data routinely collected for monitoring of program outcomes, informed consent was waived.

## Results

238 patients were admitted to the COVID Virtual Ward between 23 September to 9 November 2021, of which 58% were transferred from hospital COVID wards (early discharge) and the remaining 42% admitted directly from emergency departments or community referrals (admission avoidance).

42.4% of patients were male, 67.9% were Chinese and the mean age was 62.5 (SD 19.1), with 22.7% of patients between 70 and 79 and 21.0% of patients age 80 or over. These demographic factors did not vary significantly between the early discharge and admission avoidance groups. Compared to the admission avoidance group, the early discharge group had a higher proportion of patients who were not fully vaccinated (44.2% vs 25.0%), immunocompromised (22.4% vs 18.0%) and pregnant (7.1% vs none), however the ISARIC 4-C Mortality score was similar in both the early discharge and admission avoidance groups, with a median score of 6 (Table 2). The median length of stay in the virtual ward was 7 days in the admission avoidance group and 6 days in the early discharge group. In the early discharge group, 36 (26%) received sotrovimab and 12 (8.7%) received remdesivir prior to admission to the Virtual Ward.

**Table 2:** Demographics of patients admitted to COVID virtual ward

	Total (n = 238)	Early discharge (n = 138)	Admission avoidance (n = 100)	p-value
Age, mean (SD)	62.5 (19.1)	61.7 (19.8)	63.7 (18.0)	0.41
Age				0.73
< 50	56 (23.5)	36 (26.1)	20 (20.0)	
50–59	37 (15.5)	20 (14.5)	17 (17.0)	
60–69	41 (17.2)	22 (15.9)	19 (19.0)	
70–79	54 (22.7)	33 (23.9)	21 (21.0)	
≥ 80	50 (21.0)	27 (19.6)	23 (23.0)	
Male, n (%)	101 (42.4)	57 (41.3)	44 (44.0)	0.78
Race n(%)				0.65
Chinese	161 (67.6)	94 (68.1)	67 (67.0)	
Malay	40 (16.8)	27 (19.6)	13 (13.0)	
Indian	16 (6.7)	7 (5.1)	9 (9.0)	
Others	21 (8.8)	10 (7.2)	11 (11.0)	
Not fully vaccinated, n(%)	86 (36.6)	61 (44.2)	25 (25.0)	0.004
Immunocompromised, n(%)				
Total	49 (20.5)	31 (22.4)	18 (18.0)	0.04
Organ transplant	12 (24.4)	9 (29.0)	3 (16.7)	
Chemotherapy	20 (40.8)	11 (35.4)	9 (50.0)	
Haematological malignancy	8 (16.3)	8 (25.8)	0 (0)	
Non-cancer immunosuppression	9 (18.3)	3 (9.7)	6 (33.3)	
Pregnant, n(%)				
Total	16 (6.7)	16 (11.6)	0	NA
1st trimester	1 (6.2)	1 (6.2)	0	
2nd trimester	8 (50)	8 (50)	0	
3rd trimester	7 (44)	7 (44)	0	
Comorbidities, n(%)				0.61
0	99 (41.6)	60 (43.5)	39 (39.0)	
1	75 (31.5)	40 (29.0)	35 (35.0)	
≥ 2	64 (26.9)	38 (27.5)	26 (26.0)	
ISARIC 4C Mortality Score				
Median [IQR]	6 [3, 8]	6 [2, 8]	6 [3, 8]	0.77
0–3 (low risk), n(%)	71 (29.8)	45 (32.6)	26 (26.0)	0.224
4–8 (intermediate risk), n%()	120 (50.4)	63 (45.7)	57 (57.0)	
≥ 9 (high risk), n(%)	47 (19.7)	30 (21.7)	17 (17.0)	
Day of COVID illness at admission, median [IQR]	4 [2, 7]	6 [4, 7]	3 [2, 6]	< 0.001
LOS in virtual ward, median [IQR]	6 [3, 8]	6 [3, 8]	7 [4, 8]	0.33

41 patients (17.2%) were escalated to hospital from the Virtual Ward (Table 3). The escalation rate was 15.9% in the early discharge group and 19.0% in the admission avoidance group. The difference in these rates comprise mostly non-COVID related escalations to hospital. Patient who escalated to hospital were more likely to be older, immunocompromised and have a higher ISARIC 4C-mortality score (Additional file 1: Table S1). The majority of these escalated patients either recovered and were discharged well (56%) or returned back to COVID Virtual Ward (31.7%) when their condition stabilized.

**Table 3:** Patient Outcomes

	Total (n = 238)	Early discharge (n = 138)	Admission avoidance (n = 100)
Primary outcomes			
Escalation to hospital, n(%)	41 (17.2)	22 (15.9)	19 (19.0)
COVID-related escalation, n(%)	24 (10.1)	15 (10.8)	9 (9)
Mortality, n(%)	5 (2.1)	3 (2.1)	2 (2.0)
Secondary outcomes			
Reason for escalation, n(%)			
Hypoxia	14 (34.1)	9 (40.9)	5 (26.3)
COVID, non-hypoxic	10 (24.4)	6 (27.3)	4 (21.1)
Non-COVID medical condition	10 (24.4)	5 (22.7)	5 (26.3)
For monoclonals	4 (9.89)	2 (9.1)	2 (10.5)
Others	3 (7.3)	0	3 (15.8)
Outcome of escalation, n(%)			
Returned to virtual ward	13 (31.7)	9 (40.9)	4 (21.2)
Ward admission & recovered	19 (46.3)	10 (45.4)	9 (47.3)
ICU admission & recovered	4 (9.7)	1 (4.5)	3 (15.8)
Death	3 (7.3)	2 (9.1)	1 (5.2)
Unknown	2 (4.8)	0	2 (10.5)

The overall mortality rate was 5/238 (2.1%); all deaths were due to COVID-19 Pneumonia. 2 in the admission avoidance group (1 at home, 1 after escalation to hospital), and 3 in the early discharge group (1 at home, 2 after escalation to hospital). The three patients who were escalated to hospital were subsequently admitted to the intensive care unit prior to death. Both deaths at home were expected and the patients were provided with appropriate end-of-life care. The outcomes of 2 patients after escalation were unknown as they were admitted to a different hospital system.

All patients received at least 2 teleconsultations and 21.4% of patients received home visits for laboratory tests, COVID-19 PCR swabs and other purposes (including wound dressing, patient review, nurse education for vital signs monitoring, ECG and PICC flushing) (Table 4). There was a median of 5 teleconsults [IQR 3–7] between clinicians (doctors or nurses) and patients throughout their virtual ward admission. 24.4% of patients required at least one after-hours consult. 48.7% of patients required pharmacist consults, with a higher proportion (63% vs 29%) in the early discharge group.

**Table 4:** Service Utilisation

	Total (n = 238)	Early discharge (n = 138)	Admission avoidance (n = 100)
Vital signs monitoring, n(%)			
Automated system	185 (77.7)	106 (76.8)	79 (79.0)
Manual calls/text	53 (22.2)	32 (23.2)	21 (21.0)
Teleconsultations			
Scheduled consults per patient, median [IQR]	5 [3, 7]	4.5 [3, 7]	5 [3, 7]
Patients requiring after hour consults, n(%)	58 (24.4)	32 (23.2)	26 (26)
Patients requiring pharmacist consults, n(%)	116 (48.7)	87 (63.0)	29 (29)
Patients requiring courier services, n(%)	139 (58.4)	68 (49.3)	71 (71.0)
Patients requiring home visits, n(%)			
Total	51 (21.4)	28 (20.3)	23 (23.0)
Laboratory tests	20 (8.4)	14 (10.1)	6 (6.0)
COVID-19 PCR Swabs	13 (5.5)	9 (6.5)	4 (4.0)

77.7% of all patients engaged with the vital signs chatbot in English (85.4.1%), Mandarin (12.9%) or Malay (1.6%) (Table 5). A median of 2.4 readings were submitted per day enrolled. An average of 91% of readings had to be triggered by a push reminder, and reminders were 84% successful at triggering a vital signs submission. 72.4% or patients triggered at least one vital signs alert, 17.2% triggered for fever (temperature > 37.9 °C) and 24.8% triggered for low saturation readings (SpO_2_ < 95%).

**Table 5:** Vital signs monitoring utilisation

Category	Parameters	Total (n = 185)
Chatbot language	English, n(%)	158 (85.4)
	Mandarin, n(%)	24 (12.9)
	Malay, n(%)	3 (1.6)
Readings	Readings submitted per patient, median [IQR]	14 [8, 21]
	Readings submitted per day enrolled, median [IQR]	2.4 [2.0,2.8]
Compliance	Proportion of submissions needing reminders, mean (SD)	0.91 (0.16)
	Proportion of reminders which resulted in a reading, mean (SD)	0.84 (0.25)
Alerts	Patients who had at least one vital signs alert, n(%)	134 (72.4)
	Alerts triggered per patient, median [IQR]	3 [1.25, 5]
	Patients who triggered alert for fever, n (%)	32 (17.2)
	Patients who triggered alert for hypoxia, n (%)	46 (24.8)

Overall patient feedback was positive. 68.9% of online feedback was completed by patients, 12.5% by a household member who was also infected with COVID, 7.4% by a household member who was not infected with COVID and 7.4% by family members not living with patients (Table 6). More than 90% of respondents rated at least 4/5 for “feeling safe at home” and “how easy was it to take vital signs”. Ratings were similar across the different respondent groups, apart from those completed by household members not infected with COVID which were lower. All respondents felt that help was available if needed and would recommend the programme to others.

**Table 6:** Patient & caregiver feedback

	Total (n = 74)	Form completed by			
		Patient (n = 51)	COVID negative household member (n = 9)	COVID positive household member (n = 7)	Family not living with patient (n = 7)
Rated ≥ 4/5 for “feeling safe at home”, n(%) or N	68 (91.9)	49 (96.1)	6/9	6/7	7/7
Rated ≥ 4/5 for “easy to take vital signs”, n(%) or N	69 (93.2)	49 (96.1)	6/9	7/7	7/7
Felt help was available if needed, n(%)	74 (100)	51 (100)	9/9	7/7	7/7
Would recommend programme to others, n(%)	74 (100)	51(100)	9/9	7/7	7/7

## Discussion

In this cohort study of high-risk COVID-19 patients, admission to a technology-enabled Virtual Ward as a substitute to inpatient hospitalisation was safe and effective with high levels of patient satisfaction. The escalation to hospital rate was 17.2%, with no cases of missed or delayed deterioration resulting in collapse at home. In the admission avoidance group, 81% of patients were able to avoid hospitalisation altogether.

The rate of escalation to hospital is comparable to other published international studies (0.6–36%) [7]. Expectedly, the escalation rate was lower in the early discharge cohort after being stablisied in hospital compared to the admission avoidance cohort, however these differences were driven largely by non-COVID related escalations. The mortality rate is also comparable to other published studies (0–2.6%) [7]. Compared to studies of inpatient COVID-19 outcomes, our mortality rate was comparable or lower [26–29]. This could be explained by the differences in severity of illness, healthcare accessibility, therapeutic options and COVID variants.

Our hospital’s existing hospital-at-home programme set up the COVID Virtual Ward within 2 weeks and expanded capacity from 0 to 50 patients within 6 weeks, demonstrating the rapid scalability of hospital-at-home care models in response to clinical need. The staffing ratios, especially nursing and night requirements were generally lower than what would be required to staff an inpatient ward or equivalent community facility for such a group. This rapid scale up was enabled by the use of patients own devices for teleconsultation, and vital signs chatbot with push reminders and automated alerts, allowing larger numbers of patients to be monitored by a leaner team. In addition, without the need to build brick-and-mortar hospitals or COVID facilities [4], the key resource outlay is manpower, which is quicker to activate and train. The ability to supplement with home visits as required was also integral for the high-risk population.

We were initially concerned about the ability of patients, especially our multilingual elderly population to engage with teleconsultations and the vital signs chatbot. However, 77.7% of our patients or their caregivers engaged with the chatbot, with high compliance rates of 84%. The feature of automated reminders was useful, with an average of 91% of readings submitted only after the reminder was sent. 93% reported that the system was easy to use, however there would be a selection bias as the feedback form was also an online form. The positive feedback is similar to that seen in numerous international studies.

This study is the first description of a virtual ward for treatment of COVID-19 in Asia, including the operational model, clinical protocol, clinical outcomes, and utilisation parameters including the use of telehealth. In comparison to other virtual wards, we also describe how a vital signs chatbot and the home visit capability can expand the programme to higher risk elderly and immunocompromised patients. This study contributes to the growing literature of the use of Virtual Ward implementation as a pandemic strategy, in multiple settings.

The main limitation of this study is the absence of a control group of COVID-19 patients. Although there were no missed deteriorations in our cohort, we were unable to compare mortality and ICU admission rates, rates of hospital-acquired infection and patient satisfaction to patients who were treated in entirety in hospital or a treatment facility; nor to self-management at home without Virtual Ward care. Next, local policies regarding the criteria for isolation and hospitalisation should be considered when generalizing these findings to other settings. Furthermore, as the feedback form was distributed with discharge documents and only available in English, the patient feedback may not be representative of all patients admitted to the Virtual Ward. More studies are warranted to understand patient experiences in a Virtual Ward setting, the impact of home-based isolation and treatment on exposure and infectivity of household contacts, and comparison with hospital/institutional treatment and self-management at home.

## Conclusions

Among high-risk COVID-19 patients who are clinically stable, admission to a Virtual Ward is a safe, patient-centered and scalable alternative to inpatient hospitalisation. Healthcare financing policies should develop supporting payment strategies to enable sustainability of Virtual Wards which can quickly pivot to COVID-19 care as future pandemic waves and variants of concern emerge.

## Supplementary Information

**Additional file 1**. eAppendix.

## Data Availability

The datasets used and/or analysed during the current study are available from the corresponding author on reasonable request.

## Abbreviations

CI: Confidence interval
C: C-reactive Protein
HRP: Home Recovery Programme
INR: International normalized ratio
PICC: Peripherally inserted central catheter
