# Supplementary material for: Technology-enabled virtual ward for COVID management of the elderly and immunocompromised in Singapore: a descriptive cohort
Source: BMC Infect Dis. 2023 Feb 21;23:102. doi: 10.1186/s12879-023-08040-2 (PMC9942066; doi:10.1186/s12879-023-08040-2)
Supplement: Supplementary file 1 — Additional file 1. eAppendix. [file 12879_2023_8040_MOESM1_ESM.docx]

**eAppendix**

**Fig 1. Clinical Protocol in Virtual Ward** (N: Nurse, Dr: Doctor, TDS: three times daily, BD: twice daily, OM: once daily)


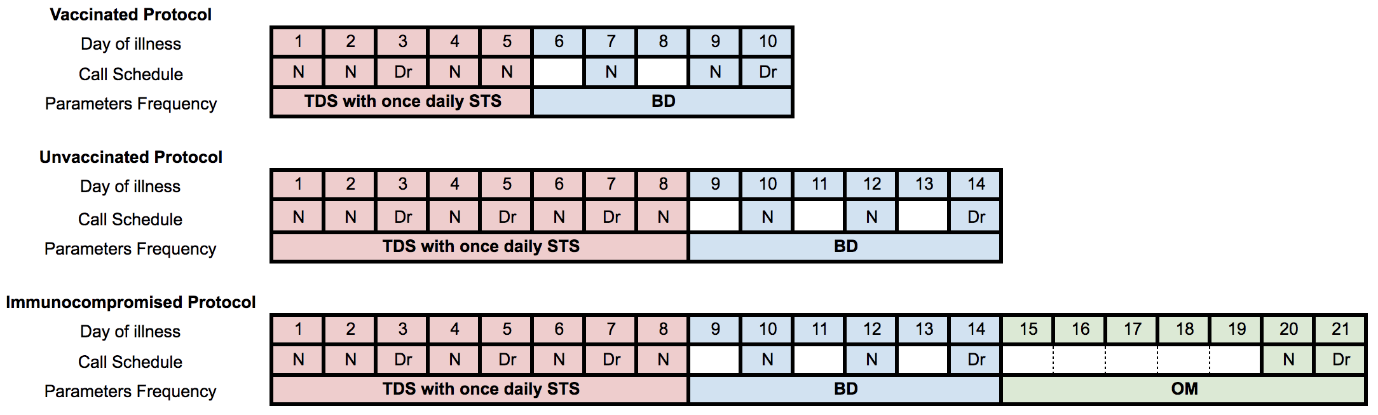


**Fig 2. Triaging Protocol for Admission Avoidance Patients**

**
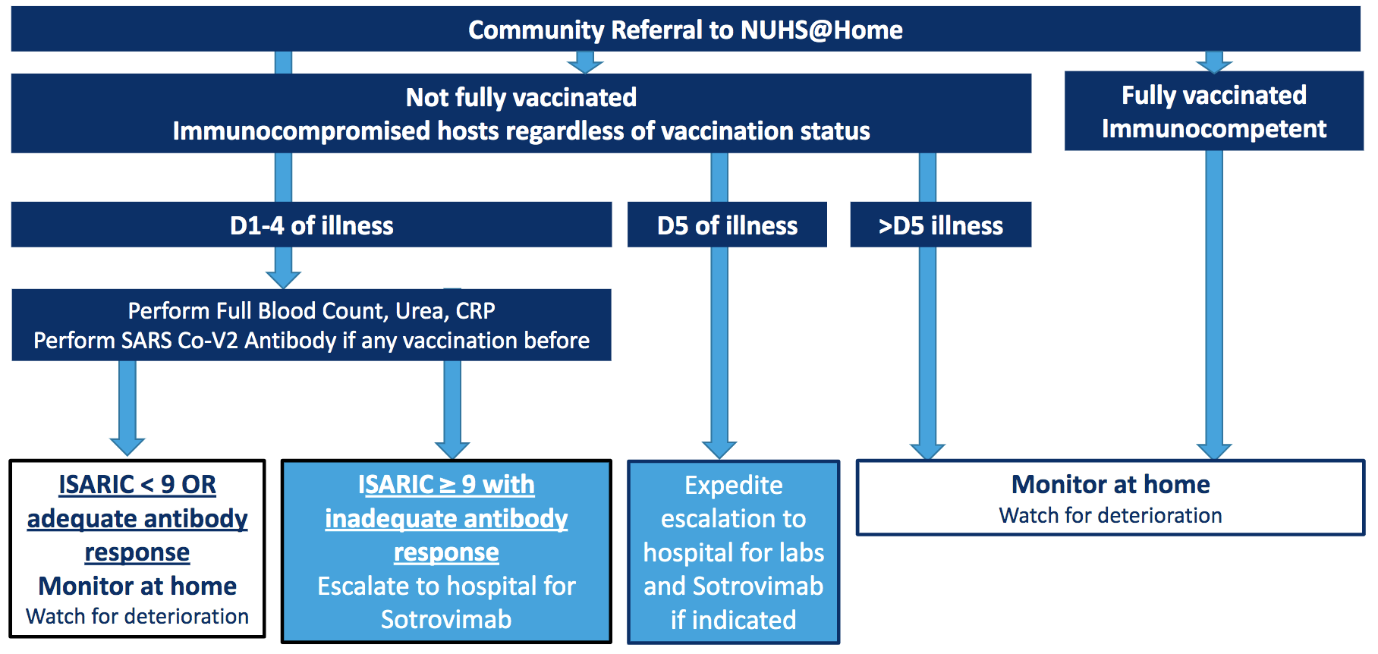
**

**eAppendix Table 1. Patients Escalated to Hospital**

**^Excludes 2 patients with expected deaths at home**

|  | **Escalated to hospital (n=41)** | **Did not require escalation to hospital (n=195)^** | **P value** |
| --- | --- | --- | --- |
| Age, mean (SD) | 66.8 (14.7) | 62.8 (19.8) | 0.18 |
| Male, n (%) | 20 (48.7) | 81 (41.5) | 0.49 |
| Race n(%)  Chinese  Malay  Indian  Others | 31 (75.6)  5 (12.2)  2 (4.8)  3 (7.3) | 130 (66.7)  35 (17.9)  14 (7.2)  16 (8.2) | 0.47 |
| Not fully vaccinated, n(%) | 14 (34.1) | 71 (36.4) | 0.17 |
| Immunocompromised, n(%)  Total  Organ transplant  Chemotherapy  Haematological malignancy  Non-cancer immunosuppression | 16 (39.0)  6 (14.6)  4 (9.7)  4 (9.7)  2 (4.9) | 33 (16.9)  6 (3.1)  16 (8.2)  4 (2.1)  7 (3.6) | <0.001 |
| Pregnant, n(%) | 2 (4.8) | 14 (7.1) | 0.74 |
| Comorbidities, n(%)  0  1  ≥2 | 17 (41.5)  15 (36.5)  9 (21.9) | 81 (41.5)  61 (31.3)  53 (27.1) | 0.81 |
| ISARIC 4C Mortality Score,  Median [IQR]  0-3 (low risk), n(%)  4-8 (intermediate risk), n%()  ≥9 (high risk), n(%) | 7 [5,9]  6 (14.6)  22 (53.6)  13 (31.7) | 6 [2,8]  65 (33.3)  97 (49.7)  33 (16.9) | 0.04 |
